# Supplementary material for: The effect of global signal regression on DCM estimates of noise and effective connectivity from resting state fMRI
Source: Neuroimage. 2020 Mar;208:116435. doi: 10.1016/j.neuroimage.2019.116435 (PMC7014820; doi:10.1016/j.neuroimage.2019.116435)
Supplement: Multimedia component 1 [file mmc1.docx]

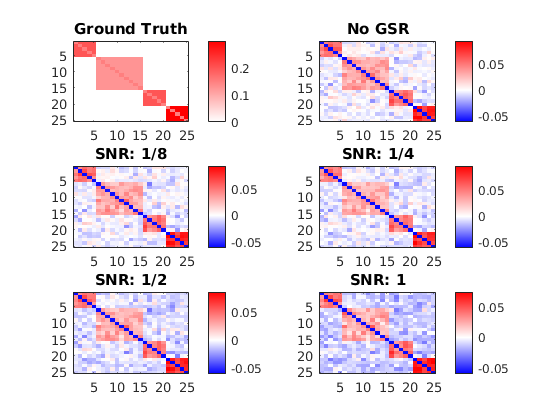

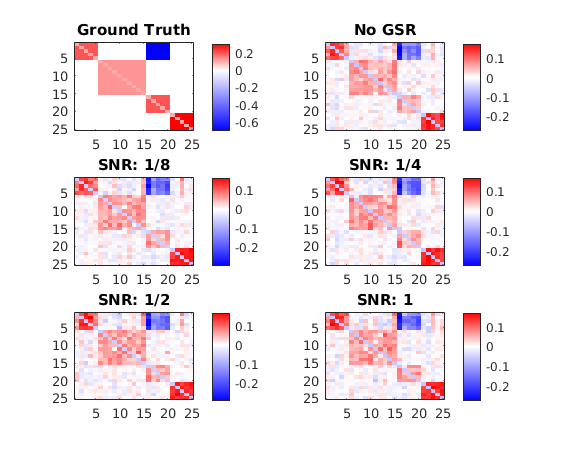


*Supplementary Figure 1.* We simulated two networks with 25 regions: One with inhibition between two networks (see left panel of figure) and one without interaction between networks (see right panel of figure). We modelled the global signal as a combination of (1) average signal from all ROIs (representing grey matter signal), (2) Gaussian noise representing a combination of noise from other sources (white matter, CSF, respiration, scanner noise, cardiac signal, …). The proportion of grey matter signal versus noise in the brain signal will in practice depend on many variables, including exact scan sequence, climate conditions, patient characteristics (aroused or not), and hardware. Therefore we looked at the effect of global signal with four different levels of noise added to the grey matter signal (exact signal to noise ratios were 1, ½, ¼, and 1/8). The left figure shows results for a network with inhibitory connectivity between two networks. As can be seen, the left network did not show spurious negative connectivity induced by GSR, while the right network showed that GSR induces some small negative connectivity between networks (which were also present but smaller in the analyses without GSR).


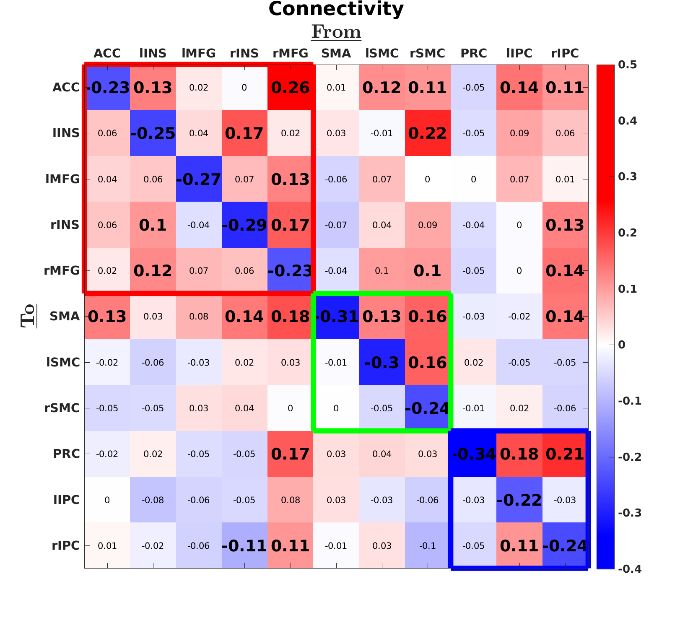

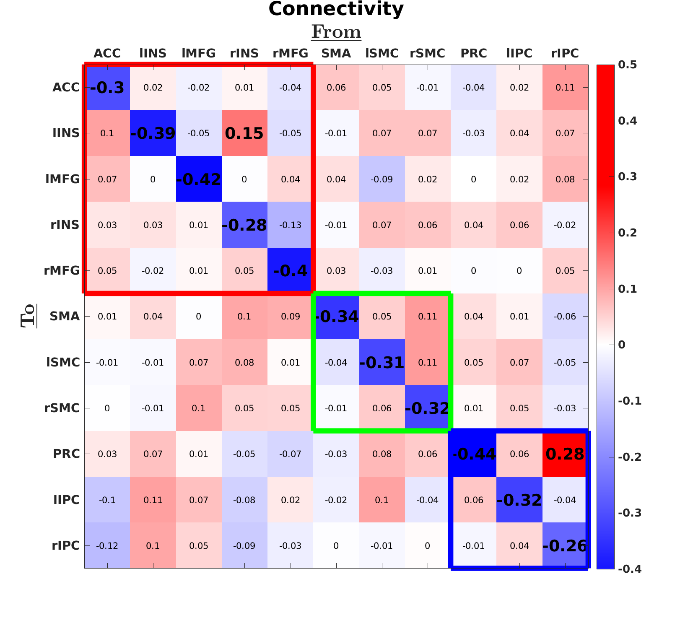

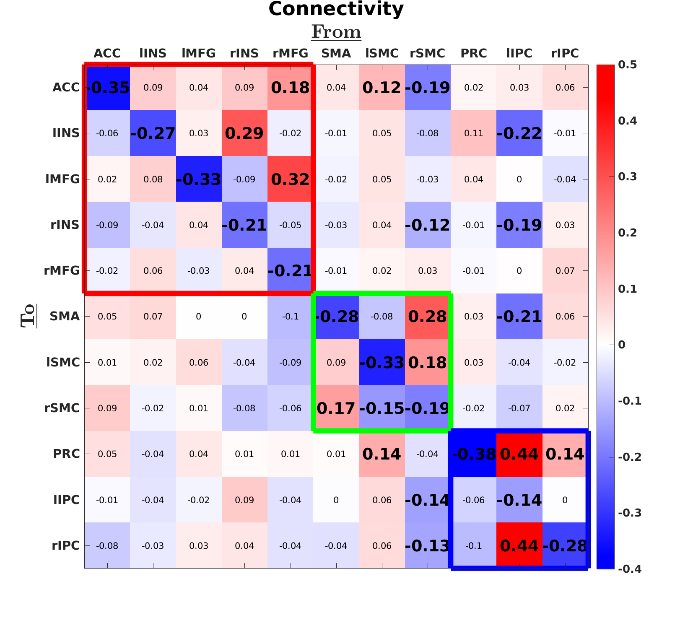

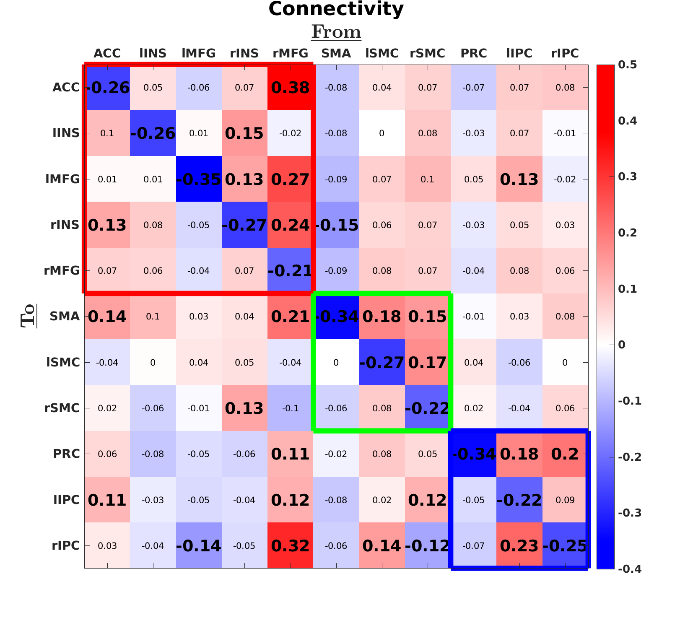

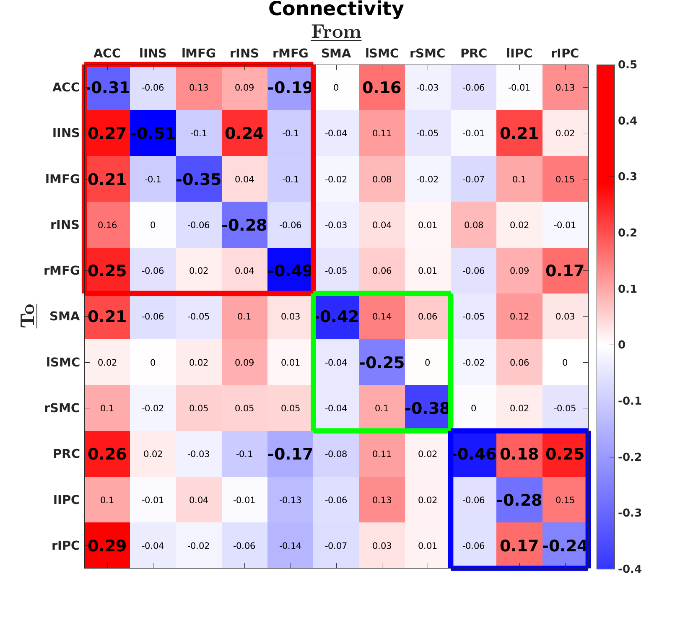

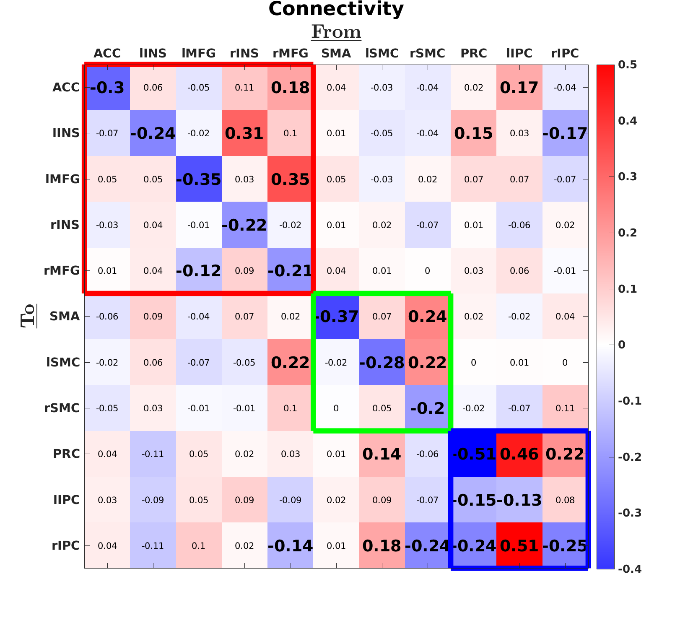


**S3**

**S1**

**S2**

**With GSR**

**Without GSR**


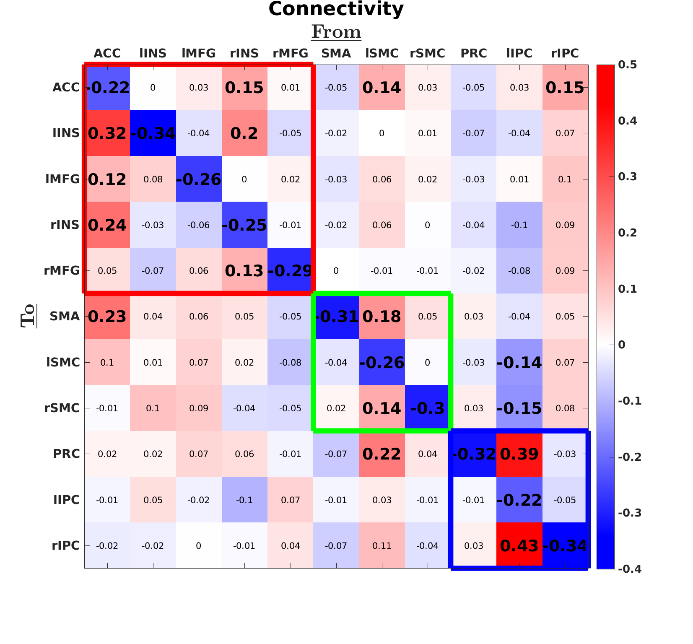

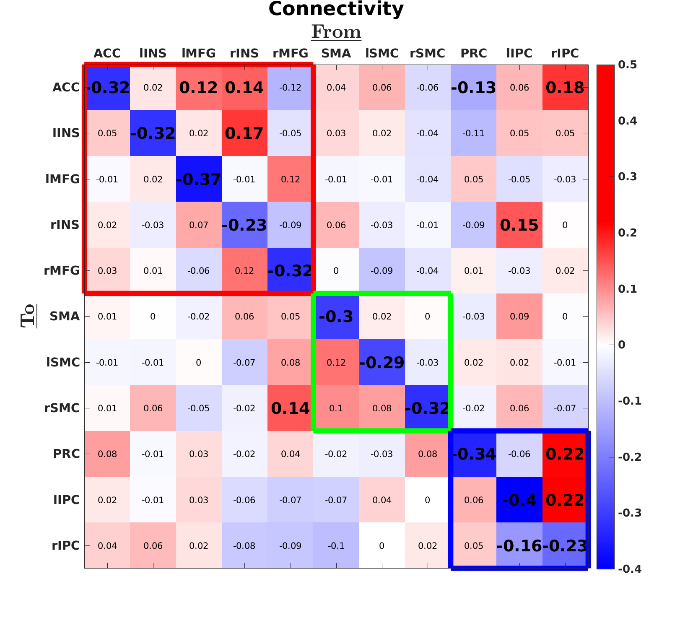

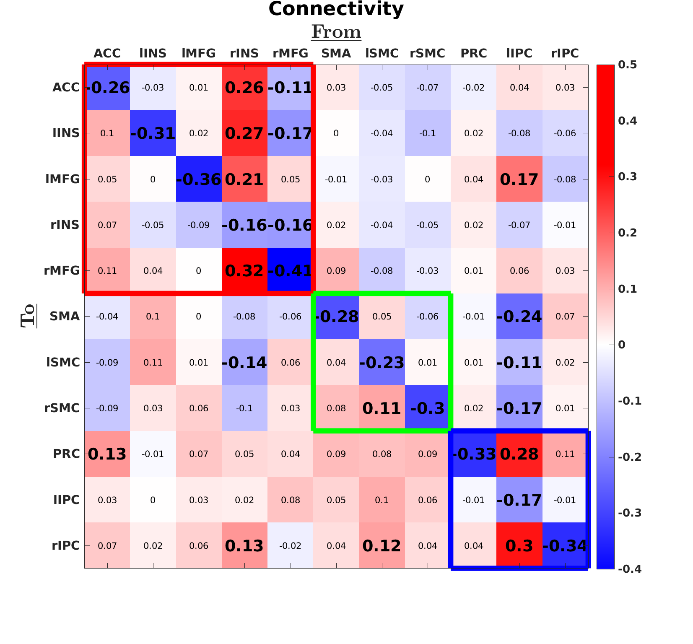

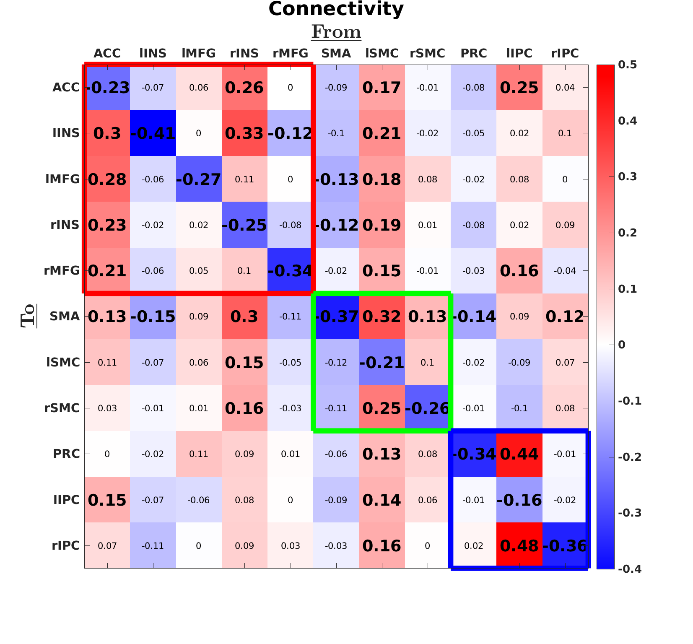

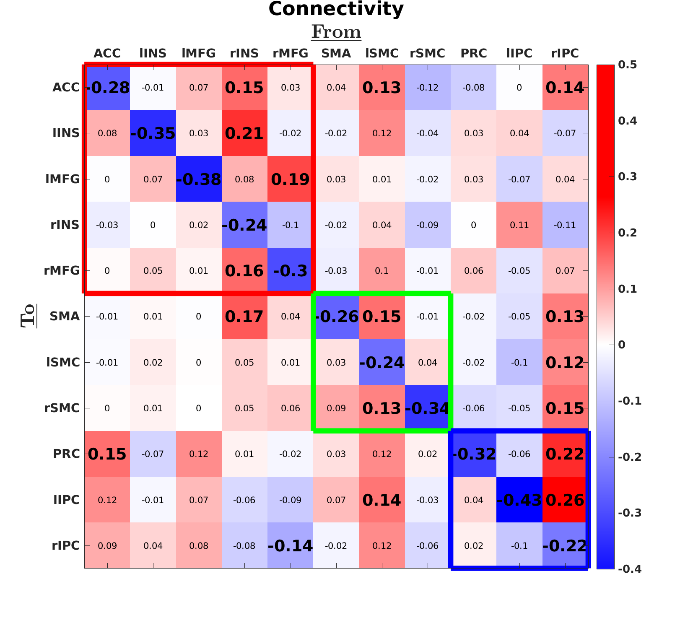

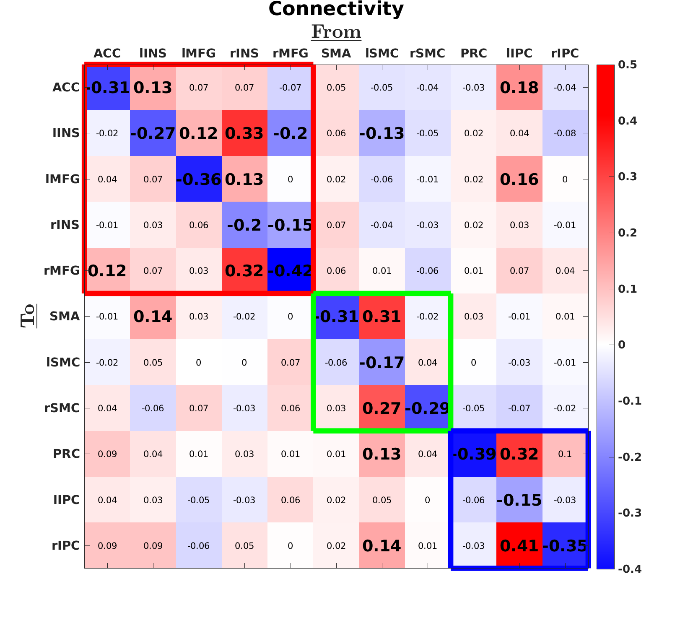


**S7**

**S6**

**S5**

**With GSR**

**Without GSR**

**With GSR**

**Without GSR**


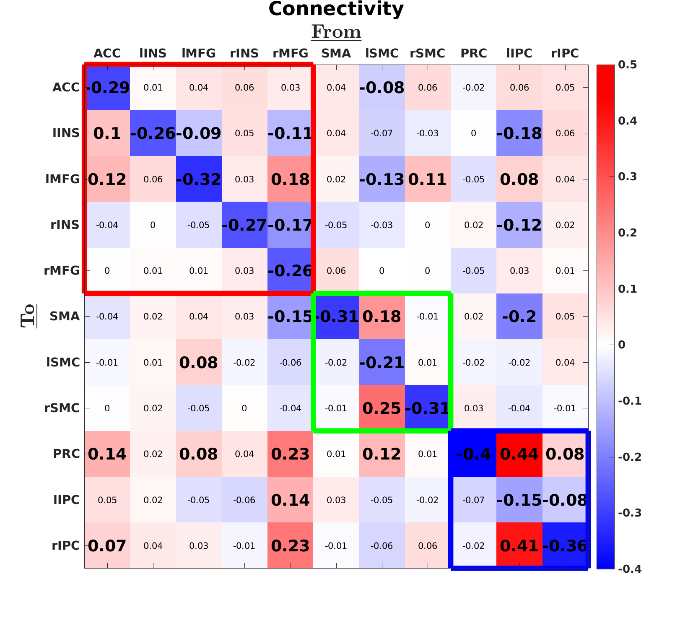

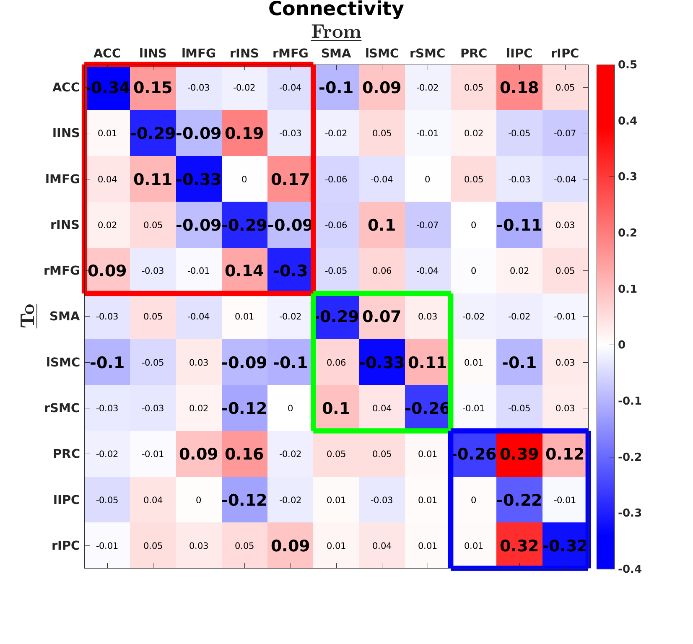

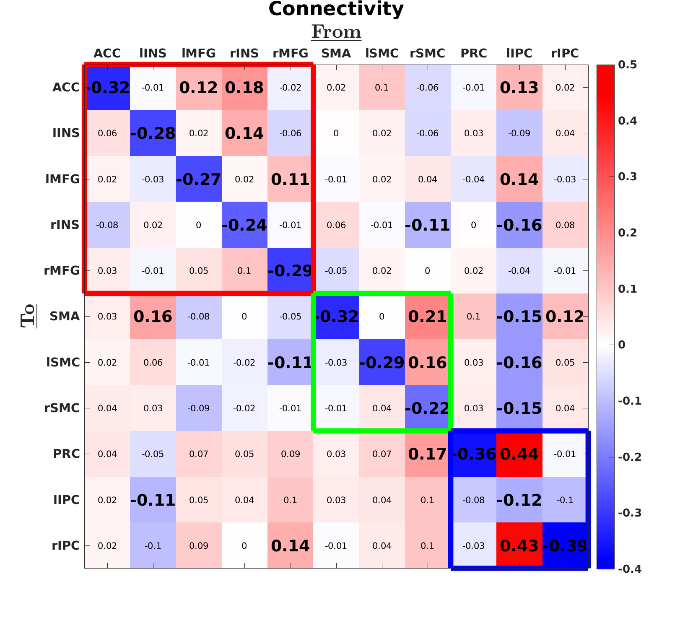

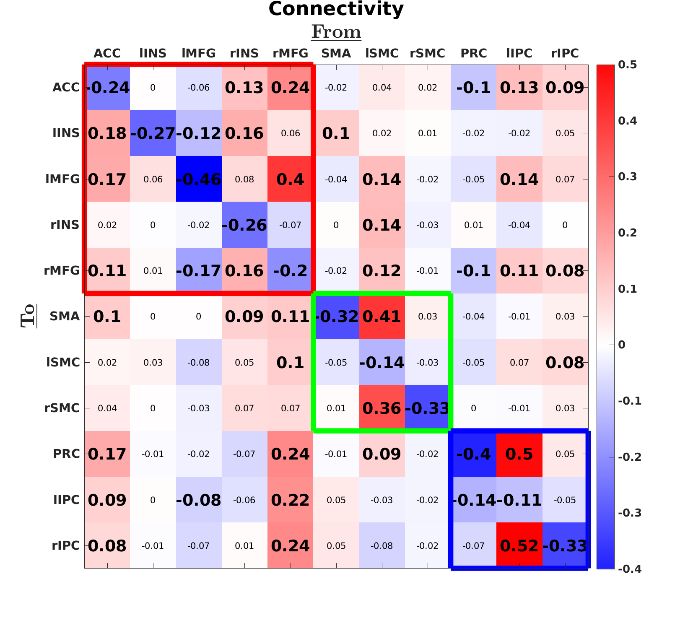

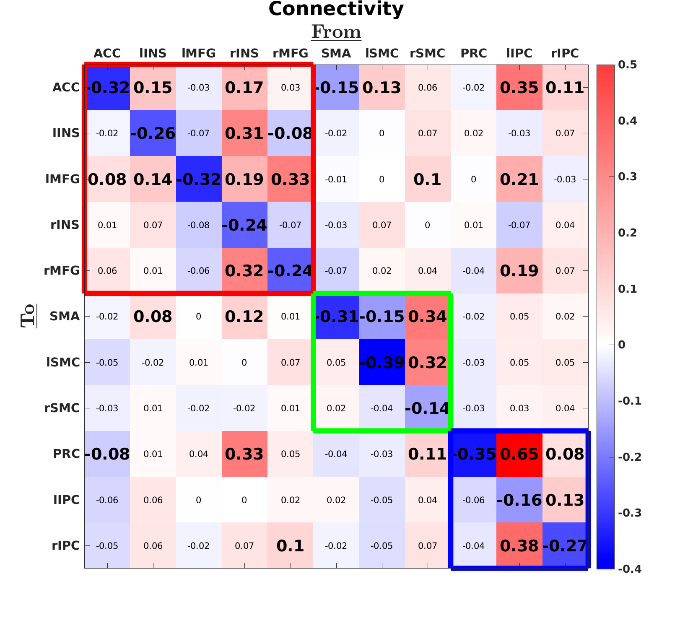

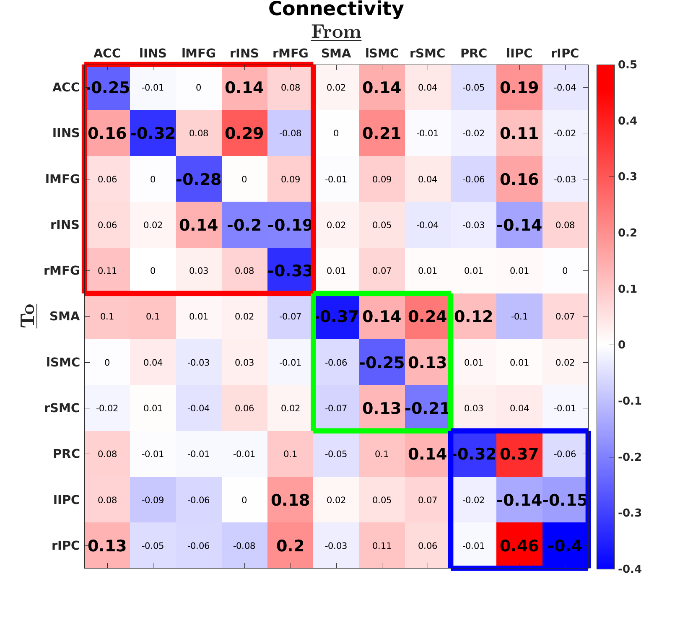


**S10**

**S9**

**S8**

**Without GSR**

**With GSR**


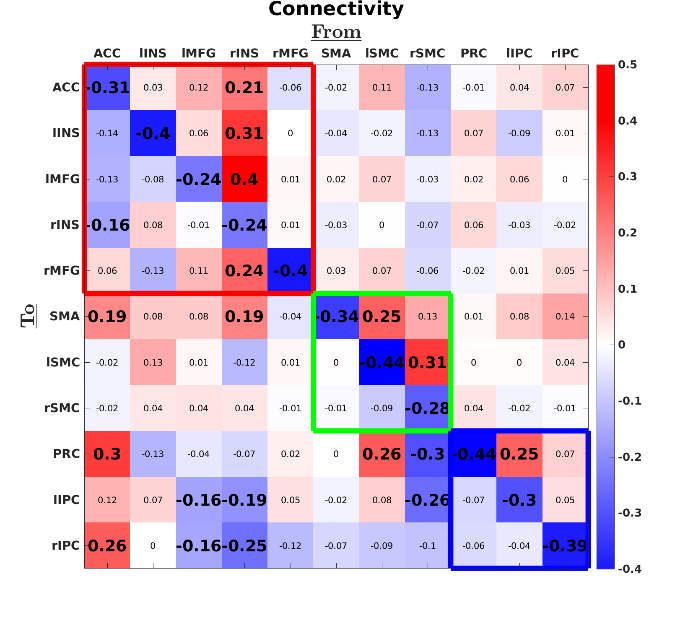

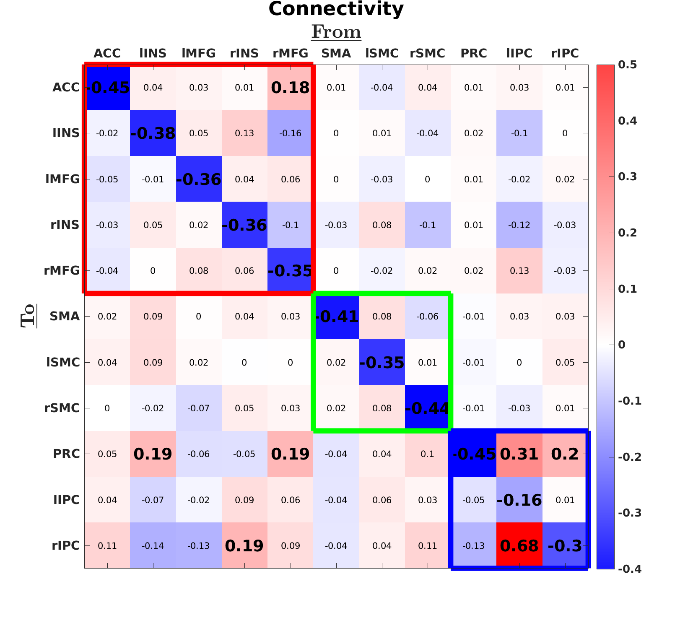

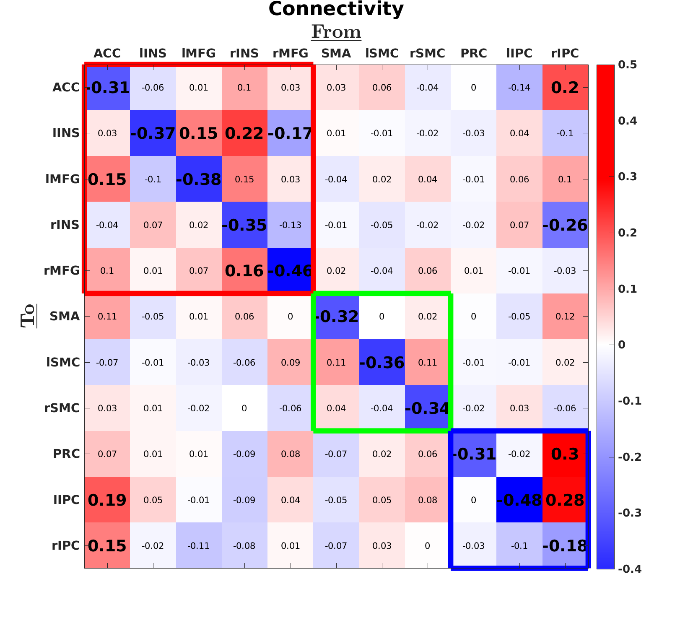

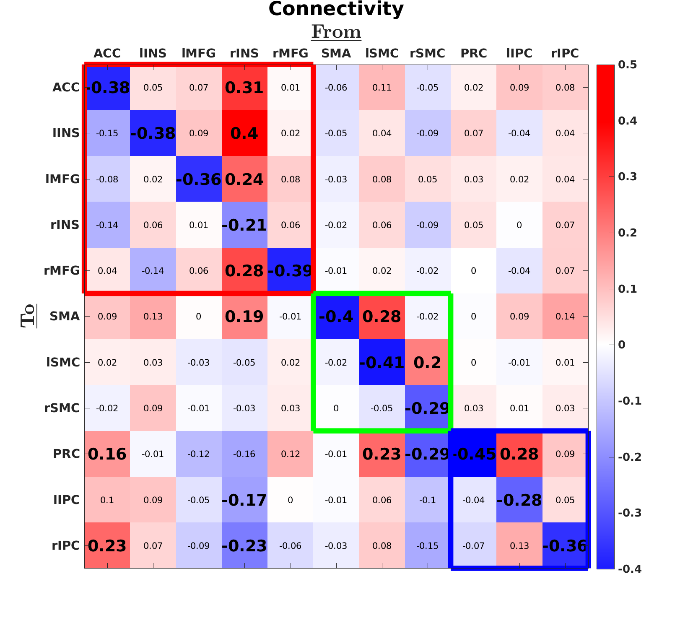

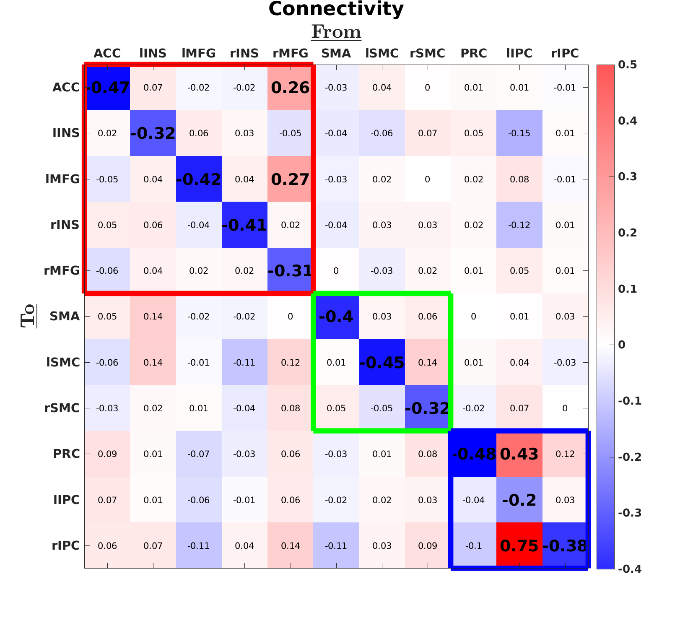

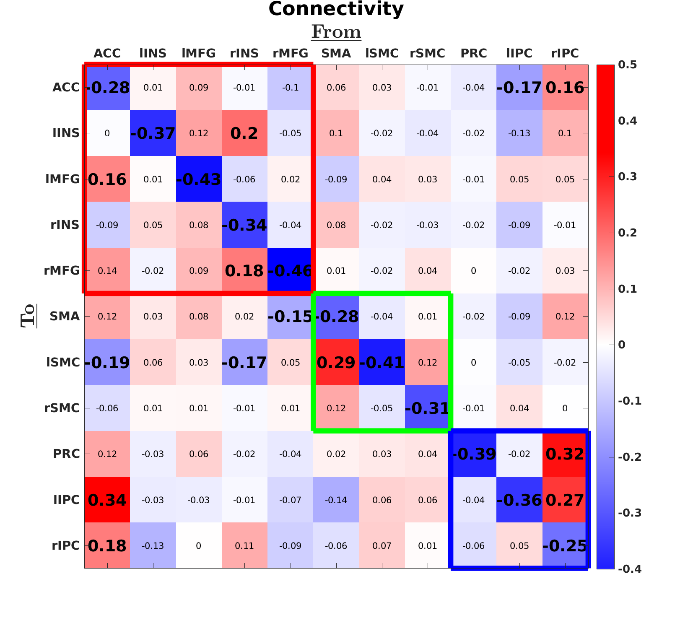


**S15**

**S12**

**S11**

**Without GSR**

**With GSR**


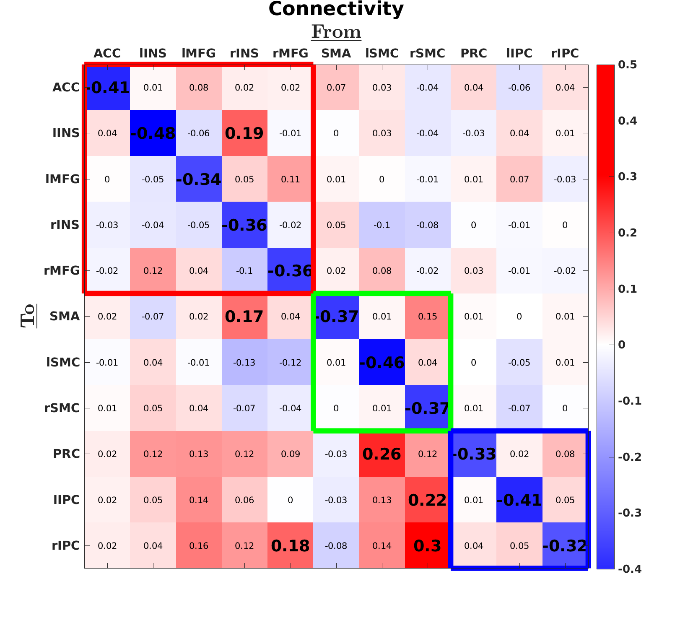

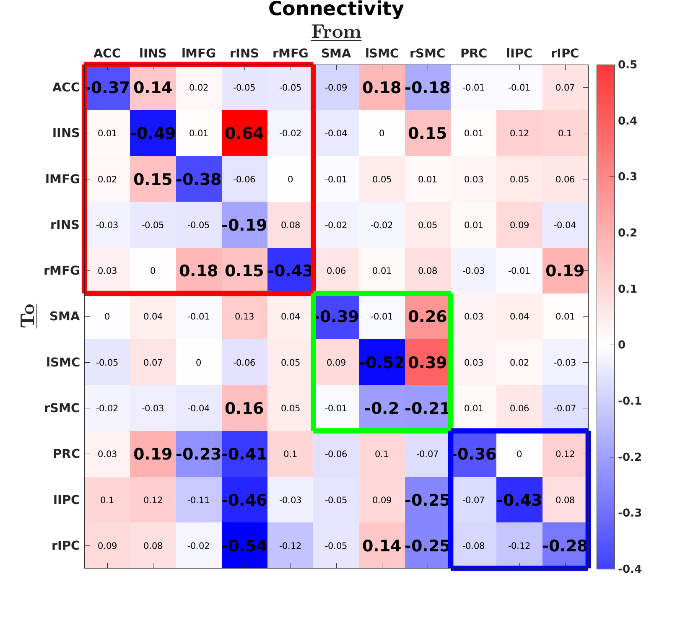

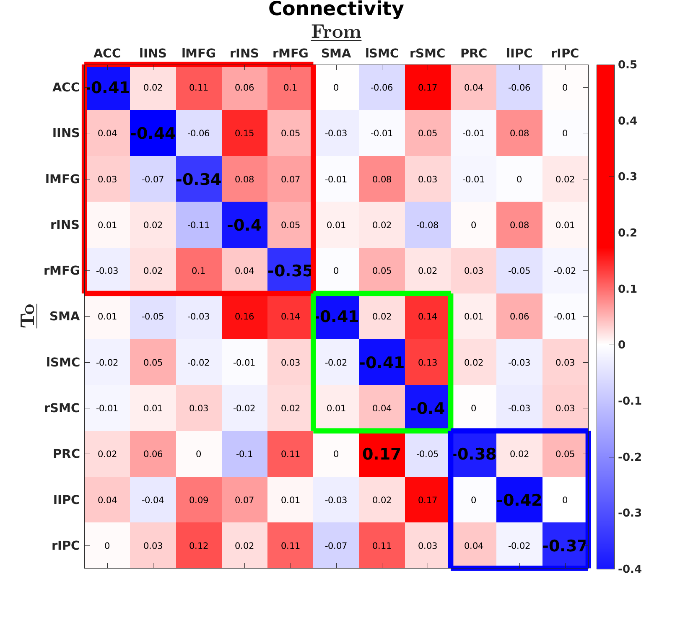

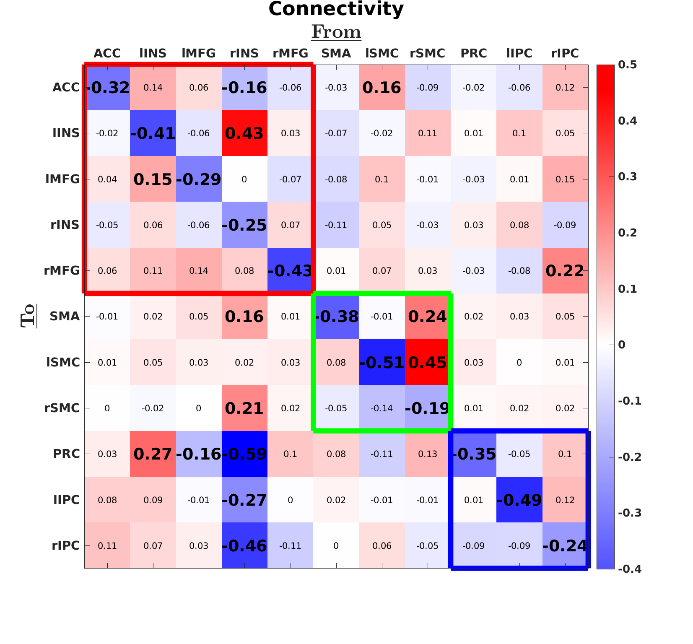


**S17**

**S16**

*Supplementary Figure 2.* Single subject connectivity patterns (1^st^ level PEB across sessions). Left column shows subject-specific connectivity patterns without GSR, right panel shows subject-specific connectivity patterns after GSR.
